# The evolving role of surface electromyography in amyotrophic lateral sclerosis: A systematic review

**DOI:** 10.1016/j.clinph.2019.12.007

**Published:** 2020-04

**Authors:** J. Bashford, K. Mills, C. Shaw

**Affiliations:** UK Dementia Research Institute, Department of Basic and Clinical Neuroscience, Maurice Wohl Clinical Neuroscience Institute, Institute of Psychiatry, Psychology and Neuroscience, King’s College, London, UK

**Keywords:** Amyotrophic lateral sclerosis, Surface electromyography, Biomarker, Motor neuron disorders, ADM, abductor digiti minimi, ALS, amyotrophic lateral sclerosis, ALS-FRS, ALS-Functional Rating Scale, APB, abductor pollicis brevis, BFS, benign fasciculation syndrome, CI, clustering index, CMAP, compound muscle action potential, FMF, fine motor function, FP, fasciculation potential, (HD)SEMG, (high-density) surface electromyography, IFI, inter-FP interval, IQR, inter-quartile range, IZ, innervation zone, MD, multiplet discharge, MFCV, muscle fibre conduction velocity, MMN, multifocal motor neuropathy, MU, motor unit, MUAP, motor unit action potential, MUNE, motor unit number estimate, MUNIX, motor unit number index, MUSIX, motor unit size index, NEMG, needle electromyography, SDTC, strength-duration time constant, SPiQE, surface potential quantification engine, SVC, slow vital capacity, TEd, depolarizing threshold electrotonus, TMS, transcranial magnetic stimulation

## Abstract

**Objective:**

Amyotrophic lateral sclerosis (ALS) is an adult-onset neurodegenerative disease that leads to inexorable motor decline and a median survival of three years from symptom onset. Surface EMG represents a major technological advance that has been harnessed in the development of novel neurophysiological biomarkers. We have systematically reviewed the current application of surface EMG techniques in ALS.

**Methods:**

We searched PubMed to identify 42 studies focusing on surface EMG and its associated analytical methods in the diagnosis, prognosis and monitoring of ALS patients.

**Results:**

A wide variety of analytical techniques were identified, involving motor unit decomposition from high-density grids, motor unit number estimation and measurements of neuronal hyperexcitability or neuromuscular architecture. Some studies have proposed specific diagnostic and prognostic criteria however clinical calibration in large ALS cohorts is currently lacking. The most validated method to monitor disease is the motor unit number index (MUNIX), which has been implemented as an outcome measure in two ALS clinical trials.

**Conclusion:**

Surface EMG offers significant practical and analytical flexibility compared to invasive techniques. To capitalise on this fully, emphasis must be placed upon the multi-disciplinary collaboration of clinicians, bioengineers, mathematicians and biostatisticians.

**Significance:**

Surface EMG techniques can enrich effective biomarker development in ALS.

## Highlights

• Surface EMG offers significant practical advantages over invasive methods in ALS patients.
• A variety of techniques exist to harness the superior spatial resolution of high-density surface EMG.
• Multi-disciplinary collaboration is required to combat analytical and technical challenges.

## 1 Introduction

Amyotrophic lateral sclerosis (ALS) is an adult-onset neurodegenerative disease of the upper and lower motor neurons, leading to inexorable motor decline and a median survival of three years from symptom onset (Al-Chalabi and Hardiman, 2013, Baumer et al., 2014). Patients demonstrate variable degrees of upper and lower motor neuron involvement and the site of symptom onset can be classified as spinal or bulbar. While the classical ALS phenotype constitutes the majority of cases (∼85%), progressive muscular atrophy (lower motor neuron involvement in isolation for > 4 years, ∼10% of cases), primary lateral sclerosis (upper motor neuron involvement in isolation for > 4 years, 1–3% of cases) and progressive bulbar atrophy (bulbar isolation, ∼2–4% of cases) make up the remainder (Al-Chalabi and Hardiman, 2013, Gordon, 2013). Anatomical isolation, a preponderance of upper motor neuron involvement and a younger age of onset confer a better prognosis.

The latest ALS diagnostic guidelines from 2008, the Awaji consensus criteria (de Carvalho et al., 2008), place a greater onus on the utility of EMG findings in an attempt to improve the diagnostic accuracy of the well-established revised El Escorial criteria (Brooks et al., 2000). Evidence of reinnervation (polyphasic, high amplitude motor units [MUs]) alongside the detection of fasciculation potentials (FPs) and/or acute denervation signs (fibrillation potentials and/or positive sharp waves), particularly in clinically strong limb muscles or bulbar muscles, confirm involvement of body regions that may go undetected by clinical examination alone. A meta-analysis showed that the sensitivity of making a diagnosis of probable/definite ALS increased from 62.2% to 81.1% when using the Awaji criteria instead of the revised El Escorial criteria, without compromising on specificity (Costa et al., 2012).

Currently, the use of needle EMG (NEMG) is routine practice in the electrodiagnosis of ALS. Concentric NEMG can evaluate the duration and amplitude of MU action potentials (MUAPs), FP duration, inter-FP interval, the number of turns and phases, the extent of intramuscular conduction block, FP variability and the presence of double discharges (Mills, 2010). Single fibre NEMG can add further estimates, including motor unit fibre density, the extent of jitter and the recency of motor unit sprouting (Janko et al., 1989). Some successful attempts have been made to further evaluate the natural history of ALS using NEMG (de Carvalho and Swash, 2013). However, due to its major disadvantage of being uncomfortable and painful for patients, serial studies tend to be avoided. In addition, the inability to reproduce the positioning of the needle on serial occasions limits NEMG as a monitoring method.

Surface EMG (SEMG) is an alternative, non-invasive method. However, in the midst of the overwhelming versatility of NEMG, the question remains as to how SEMG compares with its neurophysiological forerunner. A review conducted by the American Academy of Neurology in 2000 concluded that SEMG was inferior to NEMG in the evaluation of neuromuscular diseases in general (Pullman et al., 2000). The inability of SEMG to analyse adequately the insertional activity, interference pattern, spontaneous activity and motor unit morphology in such conditions, including ALS, represented a significant technological flaw. A subsequent evidence-based review in 2008 by the American Association of Neuromuscular and Electrodiagnostic Medicine could not find sufficient evidence to determine the clinical utility of SEMG in detecting pathological fasciculations or diagnosing ALS (Meekins et al., 2008). The committee felt inadequately informed, based on the evidence at that time, to compare the use of SEMG with established neurophysiological methods.

**Fig. 1:**
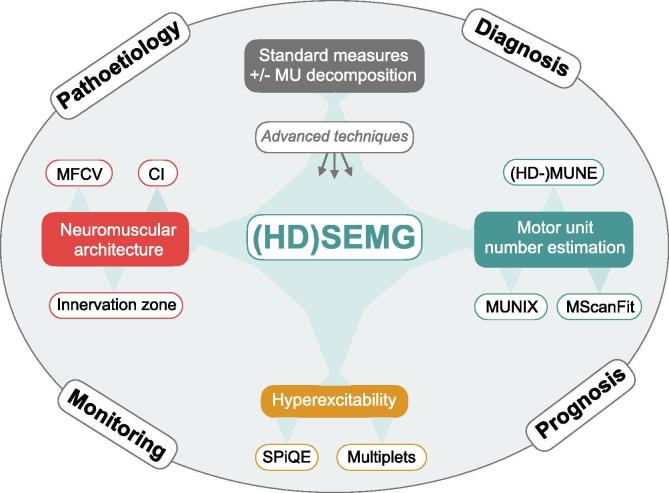
**The range of (HD) SEMG techniques in ALS.** CI, clustering index; HD-MUNE, high-density motor unit number estimate; (HD) SEMG, high-density surface electromyography; MFCV, muscle fibre conduction velocity; MScanFit, compound muscle action potential scan; MU, motor unit; MUNIX, motor unit number index; SPiQE, surface potential quantification engine.

In this review, we explore current evidence surrounding the use of SEMG in ALS patients, taking into account all study designs and all comparisons with existing measures of disease. We propose that recent advances have furnished SEMG techniques with significant advantages in the diagnosis, prognosis, monitoring and pathoetiological resolution of ALS (see Fig. 1).

## 2 Search methodology

PubMed searches with the following search terms were undertaken: ‘Amyotrophic lateral sclerosis [MeSH Terms] AND “surface EMG”’, ‘Motor neuron disease [MeSH Terms] AND “surface EMG”’, ‘Amyotrophic lateral sclerosis [MeSH Terms] AND “high-density surface EMG”’, ‘Motor neuron disease [MeSH Terms] AND “high-density surface EMG”’, ‘Amyotrophic lateral sclerosis [MeSH Terms] AND “surface electromyography”’ and ‘Motor neuron disease [MeSH Terms] AND “surface electromyography”’. In total, there were 41 unique articles published up to November 2019. One article was only available in abstract format. Seventeen articles were not reviewed as they either did not relate to ALS subjects (13), surface EMG (3) or were not in English (1 Japanese study). The remaining 23 articles were comprehensively reviewed by JB, categorising each article as having diagnostic, prognostic, disease-monitoring and/or pathophysiological relevance in human ALS subjects. Key results were extracted and summarised. Additional PubMed searches were conducted up to November 2019 to ensure full inclusion of specific techniques that incorporate surface EMG (“MUNIX”, “HD-MUNE”, “MScan”, “MScanFit”): ‘Amyotrophic lateral sclerosis [MeSH Terms] AND “*[specific technique]*”’, ‘Motor neuron disease [MeSH Terms] AND “*[specific technique]*”’. This identified an additional 19 unique primary research articles, which were reviewed by JB for relevance within the scope of this review. The reader is directed towards comprehensive reviews of motor unit number estimation (MUNE) and motor unit number index (MUNIX) elsewhere (Fatehi et al., 2018, Gawel, 2019, de Carvalho et al., 2018, Gooch et al., 2014). Other references have been cited where relevant. Authors were not contacted for additional unpublished data or clarification of results. We have reported on any techniques that made use of either single-channel or high-density surface EMG, alone or in combination with other neurophysiological methods such as peripheral nerve stimulation. Studies employing measures of upper motor neuron impairment, such as transcranial magnetic stimulation (TMS) and cortico-muscular coherence, were not covered in this review. TMS has been extensively reviewed previously (Vucic and Kiernan, 2017, Vucic et al., 2013). This systematic review was not prospectively registered on the PROSPERO database, as data extraction had already been completed when attempting to register (Page et al., 2018).

## 3 Range of SEMG techniques

### 3.1 Standard acquisition (HD) SEMG

In a similar way to NEMG, SEMG provided accurate measurements of MUAP amplitude (Milner-Brown et al., 1974), MUAP duration (Hallett, 1979), MUAP variability and morphology (Drost et al., 2007), FP frequency (de Carvalho et al., 2016, Bashford et al., 2019) and the duration required to detect a small number of FPs (Zhou et al., 2012). The maximal compound muscle action potential (CMAP) amplitude is easily captured by single-channel SEMG, however its utility as a disease biomarker has been limited. This is largely due to the stablising effect of MU reinnervation, leading to minimal changes in maximal CMAP amplitude during the early stages of ALS (Maathuis et al., 2013). More recently, CMAP decrement as a result of low-frequency repetitive nerve stimulation to the median and ulnar nerves has been demonstrated in ALS (Mori et al., 2016, Zhang et al., 2019).

**Fig. 2:**
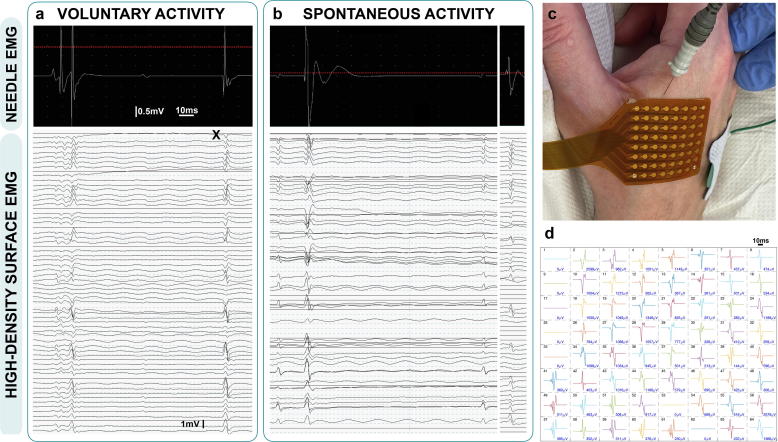
**Simultaneous needle and high-density surface EMG recordings in ALS.** Measurements were recorded from the right first dorsal interosseous (muscle power 4+/5) of an ALS patient. a. Motor unit potentials during light abduction of the index finger against resistance; b. Fasciculation potentials captured during rest; c. Experimental setup with 25 × 0.30 mm (30G) concentric needle electrode (Ambu Neuroline) and 64-channel high-density surface sensor (TMS International, Netherlands); d. Results from motor unit decomposition (Chen and Zhou, 2016) of motor unit X (seen in ‘a’) across the 64-channel array. Note channel number in top left corner of each box and peak-trough amplitude in bottom right corner for each channel. The photograph in ‘c’ has been orientated to show direct anatomical relationship with each channel in ‘d’. Time and amplitude scale bars are displayed.

High-density surface EMG (HDSEMG) incorporates multiple channels in a fixed grid formation designed to record in parallel (see Fig. 2). Automatic detection and more advanced processing of HDSEMG data has led to decomposition and classification of individual MUAPs from one recording (Jahanmiri-Nezhad et al., 2014, Jahanmiri-Nezhad et al., 2014, Jahanmiri-Nezhad et al., 2014, Chen and Zhou, 2016). This enhanced feature relied upon the improved spatial resolution achievable with HDSEMG that is not possible with NEMG or single-channel SEMG. This also provided the means to devise a clustering index (alongside two related measures), which was designed to differentiate the pattern of voluntary MUAP firing in ALS from healthy controls (Zhang et al., 2014).

### 3.2 Motor unit number estimation (MUNE)

The original MUNE method was described in 1971 (McComas et al., 1971). It was based upon incremental electrical stimulation to detect quantal jumps in the recorded CMAP. Each jump was assumed to represent the recruitment of a new motor unit. The supramaximal CMAP could then be divided by the average increase in CMAP per quantal jump in order to calculate a MUNE. This original method has led to a variety of improved techniques, such as multi-point incremental MUNE (Shefner et al., 2011) and triggered averaging techniques (Shahani et al., 1995), which have been extensively reviewed elsewhere (Gooch et al., 2014, de Carvalho et al., 2018). MUNE has been adapted by the use of HDSEMG, as the amplitudes of individual MUAPs can be averaged without the need for incremental or multi-point stimulation (Boekestein et al., 2012, van Dijk et al., 2010).

### 3.3 Motor unit number index (MUNIX) and motor unit size index (MUSIX)

MUNIX is a variation of the MUNE method, but instead of providing an estimate of the absolute number of motor units, a motor unit number index is calculated (Nandedkar et al., 2004, Nandedkar et al., 2010, Neuwirth et al., 2010, Neuwirth et al., 2015). This is a relative value, which can be compared serially within the same individual. A higher MUNIX implies a greater number of viable motor units, however some have argued that MUNIX is not a true estimate of motor unit number, relying too heavily on the maximal CMAP amplitude (Bostock et al., 2019, Nandedkar et al., 2019). MUSIX provides a measure of the average motor unit size/amplitude (Nandedkar et al., 2010). Unlike the calculation of MUNE with HDSEMG, the acquisition of MUNIX/MUSIX requires significant patient co-operation, as graded muscle contractions throughout the entire range are required. MUNIX takes 3–5 minutes per muscle to produce a result (Nandedkar et al., 2004) and has been reported to demonstrate favourable reproducibility (Neuwirth et al., 2010, Fathi et al., 2016). Good correlations between inter- and intra-operator measurements of MUNIX have been shown for both healthy controls and ALS patients across multiple centers (Nandedkar et al., 2011, Ahn et al., 2010). The latest guidelines for MUNIX assessment emphasise extra time should be taken to optimise the placement of the recording electrode, otherwise underestimates are likely (Nandedkar et al., 2018).

Most recently, the test-retest reliability of MUNIX has been assessed amongst 36 raters across 27 centres in Europe and North America prior to its inclusion as an outcome measure in one natural history study (Biogen, protocol 999AS003, ClinicalTrials.gov Identifier: NCT02611674) and one drug trial (Biogen, protocol 233AS101, ClinicalTrials.gov Identifier: NCT02623699) in ALS (Neuwirth et al., 2018). In order to qualify, each rater had to demonstrate a coefficient of variation (COV) < 20% when assessing 24 muscles on two occasions. The mean (±standard deviation) COV was 12.9% (±13.5). This study is a notable achievement on the road to implementing and standardising a validated neurophysiological biomarker across a wide geographical area.

### 3.4 MScanFit (CMAP scan)

Related to the computationally expensive Bayesian statistical method (Ridall et al., 2006), MScanFit is a more practical approach to the estimation of motor unit number. It is based upon the acquisition of multiple CMAPs at incremental stimulus intensities ranging from subthreshold to supramaximal (CMAP scan) (Bostock, 2016). Much more informative than the maximal CMAP amplitude on its own, the MScanFit model considers the full complement of active motor units, thereby reducing the sampling bias that can exist with more traditional MUNE methods. MScanFit has reported excellent inter- and intra-rater variability in the abductor pollicis brevis of ALS patients and a result can be produced in just over six minutes per muscle (Jacobsen et al., 2017).

### 3.5 Multiplet discharge (MD) detection

Some studies have used HDSEMG to detect MDs after proximal electrical stimulation in ALS patients (Sleutjes et al., 2015, Sleutjes et al., 2015, Maathuis et al., 2012). At each site, a stimulus was calibrated to activate 5–6 motor units. An MD (doublet, triplet or quadruplet) was defined as a train of identical MUAPs with inter-spike intervals <30 ms. The superior spatial resolution of HDSEMG permitted accurate characterisation of MDs into doublets, triplets or quadruplets.

### 3.6 Surface potential quantification engine (SPiQE)

This is an automated analytical method to detect and characterise fasciculations recorded by HDSEMG (Bashford et al., 2019, Bashford et al., 2020). Its design relies upon a noise-responsive algorithm, which continuously detects the noise level of the recording and adjusts the amplitude inclusion criteria for fasciculations accordingly. This was shown to achieve a favourable classification accuracy of 88% at identifying fasciculations from raw HDSEMG data.

### 3.7 Muscle fibre conduction velocity (MFCV)

SEMG has provided a non-invasive method for the calculation of MFCV (Farina and Merletti, 2004, Arendt-Nielsen and Zwarts, 1989). Although this technique has been well studied in healthy and non-ALS patients, only one study has focused on ALS patients (van der Hoeven et al., 1993).

### 3.8 Innervation zone (IZ) analysis

HDSEMG is able to provide structural information regarding the IZs of motor units supplying an individual muscle (Mesin et al., 2009, Buchthal et al., 1955, Guzman et al., 2011). A motor unit IZ length is defined as the distance between the most distal and proximal neuromuscular junctions (as detected by SEMG) in one motor unit. It has been hypothesised that as lower motor neurons die in ALS and there is subsequent reinnervation by surviving motor units, the IZ characteristics may change and therefore provide a useful marker of the disease (Jahanmiri-Nezhad et al., 2015).

## 4 Detection of key pathophysiological changes

### 4.1 Fasciculations

(HD)SEMG is a sensitive method for detecting FPs in patients with ALS (Hjorth et al., 1973, Howard and Murray, 1992, Bashford et al., 2019). It outperformed clinical observation alone over a one-minute time period, detecting FPs in 107/112 sites (95.5%) compared to 69/112 (61.6%) (Hjorth et al., 1973). A similar conclusion was made in a retrospective study of 43 ALS patients, whereby SEMG was calculated to add 4.1 (^+^/_−_ 2.4, p < 0.05) and 3.4 (^+^/_−_ 2.2, p < 0.05) sites showing fasciculations compared to clinical observation alone or conventional NEMG, respectively (Howard and Murray, 1992).

A study of 26 ALS patients showed that SEMG detected a higher frequency of FPs over a three-minute time period than clinical examination alone. At two separate anatomical sites, SEMG detected a mean FP frequency of 75.3/min (forearm extensors) and 69.7/min (forearm flexors), compared to 22.7/min and 32.7/min with clinical examination alone (Mateen et al., 2008). Most recently, SPiQE’s automated algorithm was employed to detect a median FP frequency of 65/min from biceps and gastrocnemius (Bashford et al., 2019). In some cases, large amplitude fasciculations may manifest clinically as myoclonus (Inoue et al., 2017).

### 4.2 Morphological characteristics of MUAPs

In 27 ALS patients, the MUAP duration was increased in weak biceps muscles, achieving the greatest degree of correlation in atrophic muscles (Hallett, 1979). ALS patients demonstrated the greatest degree of correlation between SEMG amplitude and force in the first dorsal interosseous muscle (Jahanmiri-Nezhad et al., 2014). Both of these changes reflect the well-established process of chronic partial denervation, a compensatory protective mechanism of MU branching in response to MU loss (de Carvalho and Swash, 2013).

## 5 Diagnostic utility

### 5.1 Distinguishing ALS from healthy controls

MDs were recorded in 94% of ALS assessments and in none of the assessments of healthy controls, suggesting that the presence of MDs may be a useful diagnostic marker in ALS (Maathuis et al., 2012). However, in a separate study, only nine out of 21 ALS patients demonstrated MDs in their thenar muscles (Sleutjes et al., 2015). Although the second study’s focus was not on the diagnosis of ALS, it implied a significant proportion of ALS patients do not demonstrate MDs. This may, in part, be due to technical reasons, as only 1.5–1.9% of electrical stimuli lead to a MD, and therefore a sufficiently large number of stimuli need to be applied before confirming their absence. The anatomical restriction (thenar muscles tested only) may also have impaired the MD detection rate in the second study.

A novel diagnostic criterion for ALS based on HDSEMG was proposed in a study of 30 ALS patients and 14 controls (Sleutjes et al., 2016). Based on 5–10 minute HDSEMG recordings of the thenar muscles at rest, potentials were characterised as either ‘isolated’ (no other potential within 250 ms), ‘continual’ (part of a train of potentials with intervals <250 ms) or ‘other’. Isolated discharges were more common in ALS patients compared to controls (35% vs. 10%, p = 0.01), as were ‘other’ discharges (33% vs. 27%, p = 0.04). Correspondingly, continual discharges were less common in ALS patients (18% vs. 54%, p = 0.003). Furthermore, the proportion of 10 s recording windows displaying at least two motor units with only isolated discharges was statistically different between ALS patients (24%, IQR 10–40%; observed in 28/30 patients) and controls (<1%, IQR 0–5%; observed in 7/14 patients). This criterion, which relies on the ability of HDSEMG to distinguish the firing of simultaneous motor units, could be a useful diagnostic marker, although confirmatory studies are required.

MScanFit demonstrated significant motor unit loss in tibialis anterior with motor unit number estimates of 45 (median; IQR 28.5–76.5) in 26 ALS patients and 117 (median; IQR 96.0–121.0; p < 0.0001) in 25 healthy controls (Kristensen et al., 2019). Receiver operating characteristic (ROC) curve analysis demonstrated the favourable classification accuracy (89%) of MScanFit MUNE at distinguishing ALS patients from healthy controls prompting future validation as a diagnostic marker. In a separate study of 35 ALS patients and 21 healthy controls MScanFit abnormalities in the abductor pollicis brevis and abductor digiti minimi of ALS patients were akin to the well-recognised “split hand” phenomenon (Sirin et al., 2019). This distinct clinical feature of ALS has also been exploited using MUNIX (Kim et al., 2016). Additionally three SEMG MUNE methods (MUNIX, MScanFit and multi-point stimulation) outperformed standard MU potential analysis using NEMG producing diagnostic accuracies between 78–89% (area under the curve) (Jacobsen et al., 2018). MUNIX has been consistently lower in ALS patients than healthy controls (Boekestein et al., 2012, Furtula et al., 2013, Fukada et al., 2016, Grimaldi et al., 2017), achieving an estimated diagnostic accuracy of 95% for 30 ALS patients and 51 healthy controls (Escorcio-Bezerra et al., 2016) and proving to have greater distinguishing power when averaged over three trials (Escorcio-Bezerra et al., 2017). Despite these positive results in limb muscles, there was no significant difference in nasalis muscle MUNIX values between ALS patients and healthy controls (Neuwirth et al., 2016).

Surface MFCV calculations from biceps brachii were greater in 22 ALS patients than healthy controls (van der Hoeven et al., 1993). The faster conducting fibres were believed to reflect the preponderance of hypertrophied muscle fibres in ALS, which develop to compensate for their atrophied neighbours. In a separate study focusing on IZ analysis (Jahanmiri-Nezhad et al., 2015), motor unit IZ lengths were increased in 12 ALS patients compared to 7 healthy controls. These two studies indicate that fundamental changes in the neuromuscular architecture of ALS patients may be a useful marker of the underlying disease process.

Using a machine learning approach, the combination of three novel HDSEMG measures, including the clustering index, distinguished ALS patients and healthy controls with 90% sensitivity and 100% specificity (Zhang et al., 2014). These measures were based on characteristic changes in voluntary MUAP features (larger, longer and more spread out potentials), known to occur as the disease progresses.

### 5.2 Distinguishing ALS from benign fasciculation syndrome (BFS)

This distinction represents a frequent problem in patients presenting with fasciculations as the prominent symptom. Two studies have directly addressed the utility of SEMG in distinguishing ALS from BFS (Kleine et al., 2012, de Carvalho et al., 2016). In the first study (Kleine et al., 2012), 15-min HDSEMG recordings of the gastrocnemius were taken from seven ALS and seven BFS patients. The focus was the detection of pairs of FPs with an inter-FP interval (IFI) between 10–110 ms. The authors proposed that an IFI of several tens of milliseconds meant the second discharge was an F-wave and therefore the first FP originated distally in the terminal arborisation (Mesrati and Vecchierini, 2004). F-waves appeared at 32 ms (BFS) and 35 ms (ALS) and the authors concluded there was no difference between the two groups.

In the second study, weak first dorsal interosseous muscles (MRC power 4/5) of ALS patients demonstrated an increase in resting fasciculation frequency from 0.25 Hz to 0.30 Hz (p = 0.013) after a sensory electrical stimulus (20 Hz, 600 stimulations) to the ipsilateral radial nerve (de Carvalho et al., 2016). Sensory stimulation caused no change in fasciculation frequency in eleven patients with BFS. This gives an indication that a difference in the spinal control mechanisms of fasciculations between these two conditions could be detected in this way.

## 6 Prognostic utility

The frequency of FPs detected by SEMG over a 3-minute period in the forearm flexors correlated positively with the degree of weakness in 26 ALS patients (Mateen et al., 2008). However, perhaps more crucially, the FP frequency was not significantly correlated with disease duration (r = 0.22, p = 0.30). Despite this, NEMG has shown higher FP frequency and shorter FP duration in strong muscles earlier in disease, indicating that comparative studies between NEMG and SEMG are warranted to fully understand the prognostic utility of fasciculations in ALS (Krarup, 2011, Bokuda et al., 2016).

In a study of 31 patients (22 with ALS, 9 with progressive muscular atrophy), a higher occurrence of MDs at baseline correlated with greater decline in the ALS-Functional Rating Scale (ALS-FRS), although this was not statistically significant (Sleutjes et al., 2016). In addition, 10/13 (77%) patients *without* MDs at baseline did not show deterioration in their fine motor function (FMF) subscore (calculated as a subset of ALS-FRS) between visits ten weeks apart, whereas 15/17 (88%) patients *with* MDs present at baseline did show deterioration in their FMF score. This points towards the potential utility of MDs as a prognostic marker, but clearly further calibration is required.

A reduction in HDSEMG-MUNE > 38% at 4 months predicted a significantly greater reduction in ALS-FRS at 8 months in a study of 18 ALS patients (van Dijk et al., 2010). This suggests this method could be calibrated to stratify patients into those with slow or fast progression at an earlier time-point. Similarly, subscores of MUNIX (MUNIXscore4 and MUNIXscore6) were useful over a six-month period to stratify patients into those with slow and fast progression (p < 0.008) (Neuwirth et al., 2015).

## 7 Use as a disease-monitoring tool

Single-channel SEMG was used to calculate MUNE in a single muscle (abductor pollicis brevis [APB]) in 21 ALS patients over a 12-month period (Felice, 1997). The decline in MUNE was similar to the decline in CMAP amplitude but greater than the decline in MRC sum power score. More recently, in 18 ALS patients, high-density MUNE declined by a greater extent compared to ALS-FRS at four months (p = 0.039) and CMAP measurement at eight months (p = 0.02) (van Dijk et al., 2010).

Longitudinal assessment of ALS patients has revealed correlations between MUNIX and ALS-FRS, CMAP and slow vital capacity (SVC) measurements (Neuwirth et al., 2010, Gawel and Kuzma-Kozakiewicz, 2016). Recording MUNIX at six muscle sites over 15 months in 51 ALS patients (Neuwirth et al., 2015), the following measures declined as indicated (mean monthly decline): ALS-FRS (2.3%), MUNIXscore6 (sum of all six muscles; 3.2%) and MUNIXscore4 (four of six muscle sites; 3.7%). Statistical correlation was only achieved in limb-onset patients from month six (p < 0.03). The greatest decline in MUNIX was seen in abductor digiti minimi (ADM) and APB muscles. However, by nine months, 17.6% of ADM and 12.1% of APB measurements has dropped to below 10% of baseline value, demonstrating a floor effect to individual muscles (Neuwirth et al., 2015). MUNIX was concluded to be comparable to MUNE in tracking disease progression, but was more convenient and less time-consuming (Boekestein et al., 2012). A separate study suggested MUNIX had more favourable intra-subject variation, similar diagnostic discernment, and improved monitoring capabilities, when compared to incremental stimulation MUNE (Furtula et al., 2013). It is noteworthy that MScanFit has demonstrated greater monthly decline over eight months (8.7% per month) than MUNIX (4.8%) (Jacobsen et al., 2019).

Notably, in patients with an upper flail-limb phenotype, MUNIX applied to the lower limbs also declined despite no clinical involvement in these limbs. The decline in MUNIX in the lower limbs outpaced the reduction in ALS-FRS or SVC and therefore is likely to provide a useful monitoring tool of pre-symptomatic limb involvement (Neuwirth et al., 2017, Fukada et al., 2016). This could be exploited in future trials of potential disease-modifying therapies as a means of assessing prevention or slowing of the disease process in subclinical anatomical regions.

Assessing the first dorsal interosseous muscle in ALS patients, single-channel SEMG did not detect a change in fasciculation frequency or amplitude over 12 months (de Carvalho and Swash, 2016). This was despite a decline in neurophysiological index (Cheah et al., 2011), indicating the degree of motor unit loss was significant over this time-frame. A significant dropout rate amongst ALS patients (only 9/34 patients underwent the fourth assessment at 12 months), analysis of a single muscle and the use of single-channel SEMG (instead of high-density SEMG) may have contributed to this negative finding.

A summary of sections 5–7 can be found in Table 1*.*

**Table 1:** The demonstrated role of (HD) SEMG techniques in ALS.

	Diagnosis	Prognosis	Monitoring
*MU decomposition*	Isolated discharges were more common in ALS^1^	Not assessed	Not assessed
*(HD-)MUNE/MUNIX*	1. MUNIX was lower in ALS than in HCs^2-5^ with estimated diagnostic accuracies of 78–95%^6–8^ 2. HD-MUNE was lower in ALS than in HCs^2^	MUNE/MUNIX stratified fast and slow progressors^9-10^	MUNE/MUNIX correlated with clinical and pre-clinical decline^2–4,9–13^
*Multiplet discharges*	MDs were more common in ALS than HCs^14–15^	Presence of MDs predicted worse clinical decline^16^	Not assessed
*Fasciculation analysis*	1. No difference in inter-fasciculation intervals between ALS and BFS^17^ 2. Fasciculation frequency increased after sensory stimulation in ALS but not in BFS^18^	Fasciculation frequency correlated with muscle weakness but not disease duration^19^	Fasciculation frequency did not correlate with clinical decline^20^
*Muscle fibre conduction* *velocity*	MFCV was increased in ALS compared to HCs^21^	Not assessed	Not assessed
*Innervation zone analysis*	IZ length was increased in ALS compared to HCs^22^	Not assessed	Not assessed
*Clustering index (with* *2 related measures)*	These measures distinguished ALS from HCs with 90% sensitivity and 100% specificity^23^	Not assessed	Not assessed

ALS, amyotrophic lateral sclerosis; BFS, benign fasciculation syndrome; HC, healthy control; IZ, innervation zone; MD, multiplet discharge; MFCV, muscle fibre conduction velocity; (HD-)MUNE, (high-density) motor unit number estimate; MU, motor unit; MUNIX, motor unit number index. References: ^1^Sleutjes et al. (2016); ^2^Boekestein et al. (2012); ^3^Furtula et al. (2013); ^4^Fukada et al. (2016); ^5^Grimaldi et al. (2017); ^6^Escorcio-Bezerra et al. (2016); ^7^Jacobsen et al. (2018); ^8^Kim et al. (2016); ^9^Neuwirth et al. (2015); ^10^van Dijk et al. (2010); ^11^Neuwirth et al. (2010); ^12^Felice et al. (1997); ^13^Neuwirth et al. (2017); ^14^Sleutjes et al. (2015); ^15^Maathuis et al. (2012); ^16^Sleutjes et al. (2016); ^17^Kleine et al. (2012); ^18^de Carvalho and Swash (2016); ^19^Mateen et al. (2008); ^20^de Carvalho and Swash (2016); ^21^van der Hoeven et al. (1993); ^22^Jahanmiri-Nezhad et al. (2015); ^23^Zhang et al. (2014).

## 8 Role in understanding the pathoetiology of ALS

SEMG has been applied to the question of where FPs and MDs originate, with the hope that this can help to clarify the underlying pathological processes in ALS. FPs can originate from the spinal portion of motor neurons, the motor axons (Kleine et al., 2008) and possibly from supraspinal areas of the central nervous system (Mills and Nithi, 1997). A study comparing fasciculations seen in ALS to those seen in BFS concluded that both pathological and benign fasciculations originated from distal portions of the nerve (Kleine et al., 2012). This correlated with findings using NEMG, which demonstrated that attempts to distinguish benign and pathological fasciculations neurophysiologically remained challenging (Mills, 2010).

A series of nerve excitability tests were performed on ALS patients and the results were compared between those who displayed MDs (as detected by HDSEMG) in the thenar muscles and those who did not (Sleutjes et al., 2015). There was no difference in the strength-time duration constant (SDTC) between the two groups (0.46 ± 0.02 ms vs 0.48 ± 0.03 ms, p = 1.00), however there were statistically significant differences in the degree of supernormality at 10 ms (25.5 ± 2.9% vs 17.0 ± 2.1%; p = 0.02) and the depolarizing threshold electrotonus (TEd) variables (TEd_peak_, 76.6 ± 2.6% vs 66.6 ± 1.7%, p = 0.001; TEd_90-100ms_, 51.7 ± 2.0% vs 44.3 ± 1.5%, p = 0.003). It is widely agreed that the SDTC value reflects the nerve’s relative excitability and is primarily correlated with the degree of membrane sodium conductance (Kanai et al., 2006). The degree of supernormality and threshold electrotonus measurements are thought to reflect underlying membrane potassium conductance, whereby a decrease in potassium conductance leads to a hyperexcitable neuron (Bostock et al., 1995, Kanai et al., 2006). Changes in both sodium and potassium conductance are considered important in the generation of fasciculations. This study, which made use of HDSEMG to detect multiplet discharges, showed evidence that MDs may share a similar mechanism, at least in part, to the generation of fasciculations (Sleutjes et al., 2015). The potential benefit of MD detection is that MDs are solely generated in the distal portions of the nerve, whereas FPs can originate anywhere from the terminal arborisation to the soma and possibly even from supraspinal regions (Maathuis et al., 2012, Kleine et al., 2008, Hirota et al., 2000). Fasciculations occur more commonly in normal individuals than MDs (Mitsikostas et al., 1998, van der Heijden et al., 1994, Kleine et al., 2008) and therefore MDs may act as a more specific marker of pathology in ALS.

## 9 Technical challenges

Significant technical challenges exist with regards to the accurate processing of potentially vast quantities of data collected by HDSEMG, where a high number of channels are recording simultaneously at necessarily high sampling rates (≥2Khz). This may be minimised to a manageable level if the recording time is reduced, but if this technology is to be used for monitoring purposes over longer time periods, then it becomes more difficult to store and analyse the data. Although the specific technical aspects of this are beyond the scope of this review, two clinical studies have so far attempted to overcome this issue in ALS patients (Jahanmiri-Nezhad et al., 2014, Bashford et al., 2019). Clinically applicable SEMG devices were designed to automatically detect FPs after amalgamation of HDSEMG data into a single average tracing. This significantly minimised the degree of offline processing and data storage required. However, this compromised the useful high-density data obtainable with HDSEMG, therefore precluding many of the techniques described in this review. Independently, SPiQE was designed to reduce the dimensionality of the HDSEMG data by focusing on the channel with the highest amplitude. This meant that the large recording volume of HDSEMG could be exploited for the purposes of identifying fasciculations without making the analysis computationally prohibitive.

Undoubtedly, future attempts to overcome these technical challenges in a clinical setting mandate the multi-disciplinary collaboration of clinicians, bioengineers, mathematicians and biostatisticians.

## 10 Complementary techniques to EMG

This review has focused on the comparison of surface and needle EMG methods, but also valuable to this discussion are the complementary techniques employed in the assessment of lower motor neuron dysfunction in ALS. These include electrical impedance myography (EIM), ultrasound and magnetic resonance imaging (MRI). EIM offers non-invasive assessment of the underlying muscle composition by passing a high-frequency, low-intensity current over a discrete area of muscle (Rutkove et al., 2007). It has been shown to track ALS progression more accurately than ALS-FRS and handheld dynamometry (Rutkove et al., 2012, Rutkove et al., 2014) and has even demonstrated value when evaluating tongue muscles (Shellikeri et al., 2015). Ultrasound can improve the detection rate of fasciculations compared to NEMG (Johansson et al., 2017, O'Gorman et al., 2017) and has shown promise as a diagnostic (Tsuji et al., 2018) and disease-monitoring tool (Vazquez-Costa et al., 2018). Fasciculation identification by ultrasound is amenable to automated quantification (Harding et al., 2016, Bibbings et al., 2019) and the development of novel SEMG sensors, transparent to ultrasound, will permit simultaneous ultrasound and SEMG recordings in future studies (Botter et al., 2019). This should provide a more comprehensive assessment of the electromechanical properties of fasciculations. Closely related to this, MRI has been used to capture MU firing during a fasciculation (Whittaker et al., 2019). Independently, short tau inversion recovery (STIR) MRI imaging of the lower limb muscles has been employed to distinguish ALS patients from spinobulbar muscular atrophy (Kennedy’s disease) and healthy controls (Klickovic et al., 2019), while higher T2 MRI signal in tibialis anterior correlated with lower MUNIX values in ALS patients longitudinally (Jenkins et al., 2018, Jenkins et al., 2019).

## 11 Conclusion

Arising from its non-invasive nature, one of the most compelling advantages of surface EMG is its practical versatility in the collection of longitudinal data. Frequent assessments of the same muscles, spanning many months to years, are necessary to capture the dynamic changes in neuromuscular architecture brought about by relentless neuronal death in ALS. Techniques that employ surface EMG without electrical stimulation, such as motor unit decomposition and SPiQE, could theoretically be applied to data collected without a clinician or technician, paving the way for remote testing in patients’ homes. This could significantly increase the intensity of data collection (e.g. weekly assessments), producing a quantity of data that is currently not achievable with hospital-based techniques and that would be amenable to powerful machine learning methods. We recommend that future research studies focus on the multi-disciplinary development of electronic hardware and automated analytical tools that are able to exploit this major advantage of surface EMG.

MUNIX has earned acceptance as a valuable outcome measure for introduction into ALS clinical trials. Although some have posited that it is not a true estimate of motor unit number, this should not deter the ongoing use of MUNIX as a well-validated biomarker that has not only demonstrated clinical correlation in multiple longitudinal cohorts but also reliability across multiple sites and users. Its translation from theory to clinical validation has been long and challenging but MUNIX is leading the charge, now fifteen years after its introduction. It serves as a valued model of biomarker development that should encourage and guide those who wish to shape the neurophysiological assessment of ALS patients in future clinical trials.

## Declaration of Competing Interest

The authors declared that there are no conflicts of interest.

## References

[b0005] Ahn S.W., Kim S.H., Kim J.E., Kim S.M., Park K.S., Sung J.J. (2010). Reproducibility of the motor unit number index (MUNIX) in normal controls and amyotrophic lateral sclerosis patients. Muscle Nerve.

[b0010] Al-Chalabi A., Hardiman O. (2013). The epidemiology of ALS: a conspiracy of genes, environment and time. Nat Rev Neurol.

[b0015] Arendt-Nielsen L., Zwarts M. (1989). Measurement of muscle fiber conduction velocity in humans: techniques and applications. J Clin Neurophysiol.

[b0020] Bashford J., Wickham A., Iniesta R., Drakakis E., Boutelle M., Mills K. (2019). SPiQE: an automated analytical tool for detecting and characterising fasciculations in amyotrophic lateral sclerosis. Clin Neurophysiol.

[b0025] Bashford J., Wickham A., Iniesta R., Drakakis E., Boutelle M., Mills K., Shaw C. (2020). Preprocessing surface EMG data removes voluntary muscle activity and enhances SPiQE fasciculation analysis. Clin Neurophysiol.

[b0030] Baumer D., Talbot K., Turner M.R. (2014). Advances in motor neurone disease. J R Soc Med.

[b0035] Bibbings K., Harding P.J., Loram I.D., Combes N., Hodson-Tole E.F. (2019). Foreground detection analysis of ultrasound image sequences identifies markers of motor neurone disease across diagnostically relevant skeletal muscles. Ultrasound Med Biol.

[b0040] Boekestein W.A., Schelhaas H.J., van Putten M.J., Stegeman D.F., Zwarts M.J., van Dijk J.P. (2012). Motor unit number index (MUNIX) versus motor unit number estimation (MUNE): a direct comparison in a longitudinal study of ALS patients. Clin Neurophysiol.

[b0045] Bokuda K., Shimizu T., Kimura H., Yamazaki T., Kamiyama T., Watabe K. (2016). Quantitative analysis of the features of fasciculation potentials and their relation with muscle strength in amyotrophic lateral sclerosis. Neurol Sci.

[b0050] Bostock H. (2016). Estimating motor unit numbers from a CMAP scan. Muscle Nerve.

[b0055] Bostock H., Jacobsen A.B., Tankisi H. (2019). Motor unit number index and compound muscle action potential amplitude. Clin Neurophysiol.

[b0060] Bostock H., Sharief M.K., Reid G., Murray N.M. (1995). Axonal ion channel dysfunction in amyotrophic lateral sclerosis. Brain.

[b0065] Botter A., Beltrandi M., Cerone G.L., Gazzoni M., Vieira T.M.M. (2019). Development and testing of acoustically-matched hydrogel-based electrodes for simultaneous EMG-ultrasound detection. Med Eng Phys.

[b0070] Brooks B.R., Miller R.G., Swash M., Munsat T.L. (2000). El Escorial revisited: revised criteria for the diagnosis of amyotrophic lateral sclerosis. Amyotroph Lateral Scler Other Motor Neuron Disord.

[b0075] Buchthal F., Guld C., Rosenfalck P. (1955). Innervation zone and propagation velocity in human muscle. Acta Physiol Scand.

[b0080] Cheah B.C., Vucic S., Krishnan A.V., Boland R.A., Kiernan M.C. (2011). Neurophysiological index as a biomarker for ALS progression: validity of mixed effects models. Amyotroph Lateral Scler.

[b0085] Chen M., Zhou P. (2016). A novel framework based on FastICA for high density surface EMG decomposition. IEEE Trans Neural Syst Rehabil Eng.

[b0090] Costa J., Swash M., de Carvalho M. (2012). Awaji criteria for the diagnosis of amyotrophic lateral sclerosis a systematic review. Arch Neurol.

[b0095] de Carvalho M., Barkhaus P.E., Nandedkar S.D., Swash M. (2018). Motor unit number estimation (MUNE): where are we now?. Clin Neurophysiol.

[b0100] de Carvalho M., Dengler R., Eisen A., England J.D., Kaji R., Kimura J. (2008). Electrodiagnostic criteria for diagnosis of ALS. Clin Neurophysiol.

[b0105] de Carvalho M., Swash M. (2013). Fasciculation potentials and earliest changes in motor unit physiology in ALS. J Neurol Neurosurg Psychiatry.

[b0110] de Carvalho M., Swash M. (2016). Fasciculation discharge frequency in amyotrophic lateral sclerosis and related disorders. Clin Neurophysiol.

[b0115] de Carvalho M., Turkman A., Pinto S., Swash M. (2016). Modulation of fasciculation frequency in amyotrophic lateral sclerosis. J Neurol Neurosurg Psychiatry.

[b0120] Drost G., Kleine B.U., Stegeman D.F., van Engelen B.G., Zwarts M.J. (2007). Fasciculation potentials in high-density surface EMG. J Clin Neurophysiol.

[b0125] Escorcio-Bezerra M.L., Abrahao A., de Castro I., Chieia M.A., de Azevedo L.A., Pinheiro D.S. (2016). MUNIX: reproducibility and clinical correlations in amyotrophic lateral sclerosis. Clin Neurophysiol.

[b0130] Escorcio-Bezerra M.L., Abrahao A., Santos-Neto D., de Oliveira Braga N.I., Oliveira A.S.B., Manzano G.M. (2017). Why averaging multiple MUNIX measures in the longitudinal assessment of patients with ALS?. Clin Neurophysiol.

[b0135] Farina D., Merletti R. (2004). Methods for estimating muscle fibre conduction velocity from surface electromyographic signals. Med Bio Eng Comput.

[b0140] Fatehi F., Grapperon A.M., Fathi D., Delmont E., Attarian S. (2018). The utility of motor unit number index: a systematic review. Neurophysiol Clin.

[b0145] Fathi D., Mohammadi B., Dengler R., Boselt S., Petri S., Kollewe K. (2016). Lower motor neuron involvement in ALS assessed by motor unit number index (MUNIX): long-term changes and reproducibility. Clin Neurophysiol.

[b0150] Felice K.J. (1997). A longitudinal study comparing thenar motor unit number estimates to other quantitative tests in patients with amyotrophic lateral sclerosis. Muscle Nerve.

[b0155] Fukada K., Matsui T., Furuta M., Hirozawa D., Matsui M., Kajiyama Y. (2016). The motor unit number index of subclinical abnormality in amyotrophic lateral sclerosis. J Clin Neurophysiol.

[b0160] Furtula J., Johnsen B., Christensen P.B., Pugdahl K., Bisgaard C., Christensen M.-K. (2013). MUNIX and incremental stimulation MUNE in ALS patients and control subjects. Clin Neurophysiol.

[b0165] Gawel M. (2019). Electrodiagnostics: MUNE and MUNIX as methods of estimating the number of motor units – biomarkers in lower motor neurone disease. Neurol Neurochir.

[b0170] Gawel M., Kuzma-Kozakiewicz M. (2016). Does the MUNIX method reflect clinical dysfunction in amyotrophic lateral sclerosis: a practical experience. Medicine (Baltimore).

[b0175] Gooch C.L., Doherty T.J., Chan K.M., Bromberg M.B., Lewis R.A., Stashuk D.W. (2014). Motor unit number estimation: a technology and literature review. Muscle Nerve.

[b0180] Gordon P.H. (2013). Amyotrophic lateral sclerosis: an update for 2013 clinical features, pathophysiology, management and therapeutic trials. Aging Dis.

[b0185] Grimaldi S., Duprat L., Grapperon A.M., Verschueren A., Delmont E., Attarian S. (2017). Global motor unit number index sum score for assessing the loss of lower motor neurons in amyotrophic lateral sclerosis. Muscle Nerve.

[b0190] Guzman R.A., Silvestre R.A., Arriagada D.A. (2011). Biceps brachii muscle innervation zone location in healthy subjects using high-density surface electromyography. Int J Morphol.

[b0195] Hallett M. (1979). Ballistic elbow flexion movements in patients with amyotrophic lateral sclerosis. J Neurol Neurosurg Psychiatry.

[b0200] Harding P.J., Loram I.D., Combes N., Hodson-Tole E.F. (2016). Ultrasound-based detection of fasciculations in healthy and diseased muscles. IEEE Trans Biomed Eng.

[b0205] Hirota N., Eisen A., Weber M. (2000). Complex fasciculations and their origin in amyotrophic lateral sclerosis and Kennedy's disease. Muscle Nerve.

[b0210] Hjorth R.J., Walsh J.C., Willison R.G. (1973). The distribution and frequency of spontaneous fasciculations in motor neurone disease. J Neurol Sci.

[b0215] Howard R.S., Murray N.M. (1992). Surface EMG in the recording of fasciculations. Muscle Nerve.

[b0220] Inoue M., Yamamoto M., Tsuzaki K., Hamano T., Etoh H., Shibasaki H. (2017). Large fasciculation can clinically manifest as spinal myoclonus; electromyographic and dynamic echomyographic studies of four cases with motor neuron disease. Clin Neurophysiol Pract.

[b0225] Jacobsen A.B., Bostock H., Fuglsang-Frederiksen A., Duez L., Beniczky S., Moller A.T. (2017). Reproducibility, and sensitivity to motor unit loss in amyotrophic lateral sclerosis, of a novel MUNE method: MScanFit MUNE. Clin Neurophysiol.

[b0230] Jacobsen A.B., Bostock H., Tankisi H. (2019). Following disease progression in motor neuron disorders with 3 motor unit number estimation methods. Muscle Nerve.

[b0235] Jacobsen A.B., Kristensen R.S., Witt A., Kristensen A.G., Duez L., Beniczky S. (2018). The utility of motor unit number estimation methods versus quantitative motor unit potential analysis in diagnosis of ALS. Clin Neurophysiol.

[b0240] Jahanmiri-Nezhad F., Barkhaus P.E., Rymer W.Z., Zhou P. (2014). Sensitivity of fasciculation potential detection is dramatically reduced by spatial filtering of surface electromyography. Clin Neurophysiol.

[b0245] Jahanmiri-Nezhad F., Barkhaus P.E., Rymer W.Z., Zhou P. (2014). Spike sorting paradigm for classification of multi-channel recorded fasciculation potentials. Comput Biol Med.

[b0250] Jahanmiri-Nezhad F., Barkhaus P.E., Rymer W.Z., Zhou P. (2015). Innervation zones of fasciculating motor units: observations by a linear electrode array. Front Hum Neurosci.

[b0255] Jahanmiri-Nezhad F., Hu X., Suresh N.L., Rymer W.Z., Zhou P. (2014). EMG-force relation in the first dorsal interosseous muscle of patients with amyotrophic lateral sclerosis. Neurorehabilitation.

[b0260] Jahanmiri-Nezhad F., Li X., Barkhaus P.E., Rymer W.Z., Zhou P. (2014). A clinically applicable approach for detecting spontaneous action potential spikes in amyotrophic lateral sclerosis with a linear electrode array. J Clin Neurophysiol.

[b0265] Janko M., Trontelj J.V., Gersak K. (1989). Fasciculations in motor neuron disease: discharge rate reflects extent and recency of collateral sprouting. J Neurol Neurosurg Psychiatry.

[b0270] Jenkins T.M., Alix J.J.P., David C., Pearson E., Rao D.G., Hoggard N. (2018). Imaging muscle as a potential biomarker of denervation in motor neuron disease. J Neurol Neurosurg Psychiatry.

[b0275] Jenkins T.M., Alix J.J.P., Fingret J., Esmail T., Hoggard N., Baster K. (2020). Longitudinal multi-modal muscle-based biomarker assessment in motor neuron disease. J Neurol.

[b0280] Johansson M.T., Ellegaard H.R., Tankisi H., Fuglsang-Frederiksen A., Qerama E. (2017). Fasciculations in nerve and muscle disorders – a prospective study of muscle ultrasound compared to electromyography. Clin Neurophysiol.

[b0285] Kanai K., Kuwabara S., Misawa S., Tamura N., Ogawara K., Nakata M. (2006). Altered axonal excitability properties in amyotrophic lateral sclerosis: impaired potassium channel function related to disease stage. Brain.

[b0290] Kim D.G., Hong Y.H., Shin J.Y., Park K.H., Sohn S.Y., Lee K.W. (2016). Split-hand phenomenon in amyotrophic lateral sclerosis: a motor unit number index study. Muscle Nerve.

[b0295] Kleine B.U., Boekestein W.A., Arts I.M., Zwarts M.J., Schelhaas H.J., Stegeman D.F. (2012). Fasciculations and their F-response revisited: high-density surface EMG in ALS and benign fasciculations. Clin Neurophysiol.

[b0300] Kleine B.U., Stegeman D.F., Schelhaas H.J., Zwarts M.J. (2008). Firing pattern of fasciculations in ALS: evidence for axonal and neuronal origin. Neurology.

[b0305] Klickovic U., Zampedri L., Sinclair C.D.J., Wastling S.J., Trimmel K., Howard R.S. (2019). Skeletal muscle MRI differentiates SBMA and ALS and correlates with disease severity. Neurology.

[b0310] Krarup C. (2011). Lower motor neuron involvement examined by quantitative electromyography in amyotrophic lateral sclerosis. Clin Neurophysiol.

[b0315] Kristensen R.S., Bostock H., Tan S.V., Witt A., Fuglsang-Frederiksen A., Qerama E. (2019). MScanFit motor unit number estimation (MScan) and muscle velocity recovery cycle recordings in amyotrophic lateral sclerosis patients. Clin Neurophysiol.

[b0320] Maathuis E.M., Drenthen J., van Doorn P.A., Visser G.H., Blok J.H. (2012). Multiplet discharges after electrical stimulation: new evidence for distal excitability changes in motor neuron disease. Amyotroph Lateral Scler.

[b0325] Maathuis E.M., Drenthen J., van Doorn P.A., Visser G.H., Blok J.H. (2013). The CMAP scan as a tool to monitor disease progression in ALS and PMA. Amyotroph Lateral Scler Frontotemporal Degener.

[b0330] Mateen F.J., Sorenson E.J., Daube J.R. (2008). Strength, physical activity, and fasciculations in patients with ALS. Amyotroph Lateral Scler.

[b0335] McComas A.J., Fawcett P.R., Campbell M.J., Sica R.E. (1971). Electrophysiological estimation of the number of motor units within a human muscle. J Neurol Neurosurg Psychiatry.

[b0340] Meekins G.D., So Y., Quan D. (2008). American Association of Neuromuscular & Electrodiagnostic Medicine evidenced-based review: use of surface electromyography in the diagnosis and study of neuromuscular disorders. Muscle Nerve.

[b0345] Mesin L., Merletti R., Rainoldi A. (2009). Surface EMG: the issue of electrode location. J Electromyogr Kines.

[b0350] Mesrati F., Vecchierini M.F. (2004). F-waves: neurophysiology and clinical value. Neurophysiol Clin.

[b0355] Mills K.R. (2010). Characteristics of fasciculations in amyotrophic lateral sclerosis and the benign fasciculation syndrome. Brain.

[b0360] Mills K.R., Nithi K.A. (1997). Corticomotor threshold is reduced in early sporadic amyotrophic lateral sclerosis. Muscle Nerve.

[b0365] Milner-Brown H.S., Stein R.B., Lee R.G. (1974). Contractile and electrical properties of human motor units in neuropathies and motor neurone disease. J Neurol Neurosurg Psychiatry.

[b0370] Mitsikostas D.D., Karandreas N., Coutsopetras P., Piperos P., Lygidakis C., Papageorgiou C. (1998). Fasciculation potentials in healthy people. Muscle Nerve.

[b0375] Mori A., Yamashita S., Nakajima M., Hori H., Tawara A., Matsuo Y. (2016). CMAP decrement as a potential diagnostic marker for ALS. Acta Neurol Scand.

[b0380] Nandedkar S.D., Barkhaus P.E., Stalberg E.V. (2010). Motor unit number index (MUNIX): principle, method, and findings in healthy subjects and in patients with motor neuron disease. Muscle Nerve.

[b0385] Nandedkar S.D., Barkhaus P.E., Stalberg E.V. (2011). Reproducibility of MUNIX in patients with amyotrophic lateral sclerosis. Muscle Nerve.

[b0390] Nandedkar S.D., Barkhaus P.E., Stalberg E.V. (2019). Motor unit number index (MUNIX) and compound muscle action potential amplitude: a reappraisal. Clin Neurophysiol.

[b0395] Nandedkar S.D., Barkhaus P.E., Stalberg E.V., Neuwirth C., Weber M. (2018). Motor unit number index: guidelines for recording signals and their analysis. Muscle Nerve.

[b0400] Nandedkar S.D., Nandedkar D.S., Barkhaus P.E., Stalberg E.V. (2004). Motor unit number index (MUNIX). IEEE Trans Biomed Eng.

[b0405] Neuwirth C., Barkhaus P.E., Burkhardt C., Castro J., Czell D., de Carvalho M. (2015). Tracking motor neuron loss in a set of six muscles in amyotrophic lateral sclerosis using the motor unit number index (MUNIX): a 15-month longitudinal multicentre trial. J Neurol Neurosurg Psychiatry.

[b0410] Neuwirth C., Barkhaus P.E., Burkhardt C., Castro J., Czell D., de Carvalho M. (2017). Motor unit number index (MUNIX) detects motor neuron loss in pre-symptomatic muscles in amyotrophic lateral sclerosis. Clin Neurophysiol.

[b0415] Neuwirth C., Braun N., Claeys K.G., Bucelli R., Fournier C., Bromberg M. (2018). Implementing motor unit number index (MUNIX) in a large clinical trial: real world experience from 27 centres. Clin Neurophysiol.

[b0420] Neuwirth C., Burkhardt C., Weber M. (2016). Motor unit number index in the nasalis muscle in healthy subjects and patients with amyotrophic lateral sclerosis. Muscle Nerve.

[b0425] Neuwirth C., Nandedkar S., Stalberg E., Weber M. (2010). Motor unit number index (MUNIX): a novel neurophysiological technique to follow disease progression in amyotrophic lateral sclerosis. Muscle Nerve.

[b0430] O'Gorman C.M., Weikamp J.G., Baria M., Van Den Engel-Hoek L., Kassardjian C., Van Alfen N. (2017). Detecting fasciculations in cranial nerve innervated muscles with ultrasound in amyotrophic lateral sclerosis. Muscle Nerve.

[b0435] Page M.J., Shamseer L., Tricco A.C. (2018). Registration of systematic reviews in PROSPERO: 30,000 records and counting. Syst Rev.

[b0440] Pullman S.L., Goodin D.S., Marquinez A.I., Tabbal S., Rubin M. (2000). Clinical utility of surface EMG: report of the therapeutics and technology assessment subcommittee of the American academy of neurology. Neurology.

[b0445] Ridall P.G., Pettitt A.N., Henderson R.D., McCombe P.A. (2006). Motor unit number estimation–a Bayesian approach. Biometrics.

[b0450] Rutkove S.B., Caress J.B., Cartwright M.S., Burns T.M., Warder J., David W.S. (2014). Electrical impedance myography correlates with standard measures of ALS severity. Muscle Nerve.

[b0455] Rutkove S.B., Caress J.B., Cartwright M.S., Burns T.M., Warder J., David W.S. (2012). Electrical impedance myography as a biomarker to assess ALS progression. Amyotroph Lateral Scler.

[b0460] Rutkove S.B., Zhang H., Schoenfeld D.A., Raynor E.M., Shefner J.M., Cudkowicz M.E. (2007). Electrical impedance myography to assess outcome in amyotrophic lateral sclerosis clinical trials. Clin Neurophysiol.

[b0465] Shahani B.T., Fang J., Dhand U.K. (1995). A new approach to motor unit estimation with surface EMG triggered averaging technique. Muscle Nerve.

[b0470] Shefner J.M., Watson M.L., Simionescu L., Caress J.B., Burns T.M., Maragakis N.J. (2011). Multipoint incremental motor unit number estimation as an outcome measure in ALS. Neurology.

[b0475] Shellikeri S., Yunusova Y., Green J.R., Pattee G.L., Berry J.D., Rutkove S.B. (2015). Electrical impedance myography in the evaluation of the tongue musculature in amyotrophic lateral sclerosis. Muscle Nerve.

[b0480] Sirin N.G., Oguz Akarsu E., Kocasoy Orhan E., Erbas B., Artug T., Dede H.O. (2019). Parameters derived from compound muscle action potential scan for discriminating amyotrophic lateral sclerosis-related denervation. Muscle Nerve.

[b0485] Sleutjes B.T., Gligorijevic I., Montfoort I., van Doorn P.A., Visser G.H., Blok J.H. (2016). Identifying fasciculation potentials in motor neuron disease: a matter of probability. Muscle Nerve.

[b0490] Sleutjes B.T., Montfoort I., van Doorn P.A., Visser G.H., Blok J.H. (2015). Diagnostic accuracy of electrically elicited multiplet discharges in patients with motor neuron disease. J Neurol Neurosurg Psychiatry.

[b0495] Sleutjes B.T., Montfoort I., van Doorn P.A., Visser G.H., Blok J.H. (2015). Increased supernormality in patients with multiplet discharges: evidence for a common pathophysiological mechanism behind multiplets and fasciculations. Clin Neurophysiol.

[b0500] Sleutjes B.T.H.M., Maathuis E.M., van Doorn P.A., Blok J.H., Visser G.H. (2016). Electrically evoked multiplet discharges are associated with more marked clinical deterioration in motor neuron disease. Muscle Nerve.

[b0505] Tsuji Y., Noto Y.-I., Shiga K., Teramukai S., Nakagawa M., Mizuno T. (2018). F48. A novel muscle ultrasound score in the diagnosis of amyotrophic lateral sclerosis. Clin Neurophysiol.

[b0510] van der Heijden A., Spaans F., Reulen J. (1994). Fasciculation potentials in foot and leg muscles of healthy young adults. Electroencephalogr Clin Neurophysiol.

[b0515] van der Hoeven J.H., Zwarts M.J., van Weerden T.W. (1993). Muscle fiber conduction velocity in amyotrophic lateral sclerosis and traumatic lesions of the plexus brachialis. Electroencephalogr Clin Neurophysiol.

[b0520] van Dijk J.P., Schelhaas H.J., van Schaik I.N., Janssen H.M., Stegeman D.F., Zwarts M.J. (2010). Monitoring disease progression using high-density motor unit number estimation in amyotrophic lateral sclerosis. Muscle Nerve.

[b0525] Vazquez-Costa J.F., Campins-Romeu M., Martinez-Paya J.J., Tembl J.I., Del Bano-Aledo M.E., Rios-Diaz J. (2018). New insights into the pathophysiology of fasciculations in amyotrophic lateral sclerosis: an ultrasound study. Clin Neurophysiol.

[b0530] Vucic S., Kiernan M.C. (2017). Transcranial magnetic stimulation for the assessment of neurodegenerative disease. Neurotherapeutics.

[b0535] Vucic S., Ziemann U., Eisen A., Hallett M., Kiernan M.C. (2013). Transcranial magnetic stimulation and amyotrophic lateral sclerosis: pathophysiological insights. J Neurol Neurosurg Psychiatry.

[b9010] Whittaker R.G., Porcari P., Braz L., Williams T.L., Schofield I.S., Blamire A.M. (2019). Functional magnetic resonance imaging of human motor unit fasciculation in amyotrophic lateral sclerosis. Ann. Neurol..

[b0540] Zhang D., Zhao Y., Yan C., Cao L., Li W. (2019). CMAP decrement by low-frequency repetitive nerve stimulation in different hand muscles of ALS patients. Neurol Sci.

[b0545] Zhang X., Barkhaus P.E., Rymer W.Z., Zhou P. (2014). Machine learning for supporting diagnosis of amyotrophic lateral sclerosis using surface electromyogram. IEEE Trans Neural Syst Rehabil Eng.

[b0550] Zhou P., Li X., Jahanmiri-Nezhad F., Rymer W.Z., Barkhaus P.E. (2012). Duration of observation required in detecting fasciculation potentials in amyotrophic lateral sclerosis using high-density surface EMG. J NeuroEng Rehabil.

